# Association between dietary phytochemical index and risk of benign prostatic hyperplasia: a case-control study

**DOI:** 10.1186/s41043-024-00531-5

**Published:** 2024-03-01

**Authors:** Seyedeh Neda Mousavi, Maryam Nouri, Esmaeil Yousefi Rad, Reza Kazemi, Mehdi Birjandi, Shelly Coe, Somayeh Saboori

**Affiliations:** 1 Zanjan Metabolic Diseases Research Center, Zanjan University of Medical Sciences, Zanjan, Iran; 2 Department of Nutrition, School of Medicine, Zanjan University of Medical Sciences, Zanjan, Iran; 3 Student Research Committee, Lorestan University of Medical Sciences, Khorramabad, Iran; 4 Oxford Brookes Centre for Nutrition and Health (OxBCNH), Department of Sport, Health Sciences and Social Work, Faculty of Health and Life Sciences, Oxford Brookes University, Oxford, UK; 5 Nutritional Health Research Center, School of Health and Nutrition, Lorestan University of Medical Sciences, Khorramabad, Iran; 6 Department of Urology, Al-Zahra Hospital, Isfahan University of Medical Sciences, Isfahan, Iran

**Keywords:** Phytochemicals, DPI, Benign prostatic hyperplasia, Diet, Case-control

## Abstract

**Background:**

Dietary intake of phytochemicals has been associated with a reduced risk of chronic diseases, but research on their relationship with benign prostatic hyperplasia (BPH) is limited. This case-control study aimed to investigate the association between a Dietary Phytochemical Index (DPI) and BPH risk in a Middle-Eastern population.

**Methods:**

The study recruited 112 BPH patients and 112 age-matched healthy controls (40–75 years) from Al-Zahra Hospital Clinic in Isfahan, Iran between 2021 and 2022. Dietary intake was assessed using a validated food-frequency questionnaire, and DPI was calculated as the ratio of energy intake from phytochemical-rich foods to total daily energy intake. Logistic regression analysis was performed, adjusting for potential confounders.

**Results:**

In the crude model, participants in the highest DPI tertile had a 70% lower odds of BPH compared to those in the lowest tertile (OR:0.3, 95% CI 0.15–0.61, P-trend = 0.001). After adjusting for confounders, this inverse association remained significant (OR:0.23, 95% CI 0.15–0.63, P-trend = 0.001). Participants with higher DPI consumed more whole grains (*p* = 0.02), nuts (*p* < 0.001), legumes (*p* = 0.02), fruits (*p* < 0.001), vegetables (*p* < 0.001), olives and oilve products (*p* = 0.02), and tomato and its products (*p* < 0.001) in their diet compared to the lowest tertile. However, red meat (*p* = 0.03) and refined grains (*p* < 0.001) were consumed in higher amounts in the lowest tertile compared to the highest DPI tertile.

**Conclusions:**

This study demonstrates a protective association between DPI and BPH risk in the Middle-Eastern population. Encouraging higher intake of phytochemical-rich foods may help reduce the risk of BPH, highlighting the relevance of nutritional science in promoting prostate health.

## Introduction

Benign prostatic hyperplasia (BPH) is an increasing concern in aging men and involves the lower urinary tract [1]. Hyperplastic nodules are formed in the periurethral region and transition duct of the prostate, enlarging to the urethra. The storage and voiding symptoms include nocturia, weak urinary stream, and incomplete bladder emptying, straining to void and an intermittent stream of urine produced [2, 3]. Recent evidence has shown that aside from age, metabolic factors including metabolic syndrome, hormonal imbalances including sex steroid hormones and vitamin D levels, are all associated with an increased risk of BPH [4–7]. Obesity, especially central obesity, and also dyslipidemia, hypertension and poor glycemic control are the main modifiable metabolic risk factors for BPH [8–11]. Epidemiological studies report the potential effect of diet on the incidence and development of prostatic diseases [12–15]. In patients with prostatic cancers, the protective effect of a Mediterranean diet has been shown due to the high anti-oxidant and polyphenol content of the diet [16]. Moreover, a decrease in protein intake from animal sources and inclusion of fruits and vegetables in high amounts may also play a protective role in BPH [17]. There has been a growing focus on the production and utilization of medicinal plants, which serve as potential sources of natural bioactive compounds, for the treatment of various prostate diseases, including benign hypertrophy, prostatitis, and chronic pelvic pain syndrome [8, 18]. Phytochemicals are one of the bioactive group of molecules in these plants that show potential as therapeutic agents due to their anti-oxidant and anti-inflammatory properties [18–20]. The Dietary Phytochemical Index (DPI) is a quantitative index defined as the percentage of energy intake derived from foods rich in phytochemicals [21]. A number of previous studies investigated the association between DPI and chronic diseases including breast cancer [22], hypertension [23], and Type 2 Diabetes [24]. To our knowledge, the effect of phytochemical intake on the risk of BPH development has not been assessed so far. Considering the importance of dietary components as the chronic effective factors on health, the present study compared the DPI in newly diagnosed patients with BPH with that in healthy men.

## Methods

### Participants

The current case-control study was carried out on 112 newly-identified cases of BPH and 112 healthy-controls between the ages of 40 and 75 years in Isfahan Al-zahra Hospital Clinic, Iran, between 2021 and 2022. Sample size calculation for study was performed using the formula for calculating sample size in a logistic regression model: n= [(Z^2^_α/2_​*P*(1 − P)*k)/ES^2^ ]. Where Z _α/2_ (for a significance level of 0.05) ≈ 1.96, P (estimated proportion of the outcome) = 0.23 [25], assumed ES (effect size) = 0.78 [26], k ≈ 15, Ratio of Cases to Controls = 1:1, The sample size was initially calculated to be 107 for each group. To accommodate potential 10% data loss, an additional 5 patients were included in each group. Consequently, a total of 112 patients were recruited for each group. The participants were selected using a consecutive random sampling method. Prior to data collection, each participant signed a written informed consent form. Prior to recruitment an experienced urologist diagnosed BPH patients by the use of the patient’s history, digital rectal examination (DRE), and laboratory results, such as serum prostate-specific antigen (PSA between 4 and 10 ng/mL). We also evaluated the results of the prostate biopsy and excluded patients with PSA levels > 10 ng/mL in order to exclude prostatic cancer in suspicious cases. BPH patients suspected of having prostate cancer underwent a series of clinical and diagnostic investigations, which included additional assessments such as MRI-Ultrasound Prostate Fusion Biopsy, or saturation biopsies if deemed necessary by the urologist. Patients who had been diagnosed with BPH, no longer than 6 months before the study, were offered the chance to take part and if consented, were included in the study. Those who had a prostatectomy, a history of prostate cancer or any other malignancies, urinary tract infection or intractable urinary retention, chronic diseases including any type of diabetes, liver, thyroid, cardiovascular diseases, and kidney diseases, anyone following a special diet, use of appetite suppressants or anti-obesity drugs, use of multivitamin and mineral supplements were excluded from the study. The controls were also randomly selected from the same Clinic by a urologist with the same criteria as cases but had no history of BPH or prostate cancer and were visited the clinic for a routine appointment or were hospital attendees and they were requested to consult the urologist to verify that they had not been diagnosed with BPH. We matched the case and control participants within 5-year age groups in order to increase comparability among the study groups. The effects of confounding variables such as body mass index (BMI), educational level, physical activity, waist circumference, smoking status, marital status and dietary intake of red meat, fish, cholesterol, egg, coffee, total fat and refined grains were adjusted. The study’s protocol and design were approved by the ethics committee of Lorestan University of Medical Sciences in accordance with the ethics code IR.LUMS.REC.1400.081.

### Dietary assessment

The dietary intake of participants was evaluated by a trained nutritionist using the Iranian valid and reliable 168-items semi-quantitative food frequency questionnaire (FFQ) with the standard serving sizes commonly consumed by Iranians [27]. Individuals were asked about their past year of food consumption frequency on a daily, weekly, and monthly basis. Each food type’s reported quantities were converted to grams per day. By multiplying the daily frequency of intake by the nutrient content of the specified portion size, daily energy and nutrient consumption was determined using the Nutritionist IV software which is based on the U.S. Department of Agriculture’s food composition database (modified for Iranian foods) [28].

### Other variables

Each participant was asked to report their age, education, marital status, smoking habits, family history of BPH and medical history. A digital scale (accuracy of 100 g) and a stadiometer (accuracy of 0.1 cm) were used to measure the participants’ weight and height, respectively via standard methods. BMI was collected and reported. To the nearest 0.1 cm, waist circumference was measured with a plastic measuring tape at the midpoint between the lowest rib cage and above the iliac crest. The valid and reliable Iranian version of the International Physical Activity Questionnaire (IPAQ) was used to assess physical activity [29]. The Metabolic Equivalent (MET) of physical activity was calculated and categorized in to three following group: Light Activity (MET < 3.0), Moderate Activity (MET 3.0-5.9)),Vigorous Activity (MET ≥ 6.0) [30].

### Calculation of DPI score

To calculate the DPI, we used the McCarty equation as follows [21] [Daily energy intake from phytochemical-rich foods (kcal)/ Total daily energy intake (kcal)] *100. In the current study, the following phytochemical-rich foods were taken into consideration: Whole grains, fruits (orange, yellow, and red); vegetables (including starchy vegetables, dark green vegetables, orange vegetables, and red vegetables); products made from soybeans; nuts (pistachio, hazelnut, almond, walnut, and peanut); legumes (chickpeas, beans, and lentils); olives; olive oil, juices from natural fruits and vegetables (carrot, orange, and lemon). Potato as a vegetable food was not included in the DPI calculation because of its low phytochemical content.

### Statistical analyses

The Kolmogorov–Smirnoff test was applied to examine the distribution of data related to normality. Tertile ranges of DPI scores were assigned for classification of participants. The Chi-square test was used to investigate categorical variables across DPI tertiles. A One-Way Analysis of Variance (ANOVA) was used to evaluate differences in continuous variables between DPI tertiles. For variables that were not normally distributed, either the Mann–Whitney U test or the Kruskal–Wallis test was used. The relationship between DPI and BPH was evaluated using binary logistic regression. Age, energy intake (kcal/day), physical activity levels (light/ moderate/vigorous), family history of BPH (yes/no), marital status (yes/no), education, waist circumference, smoking status (smoker/nonsmoker), BMI, and dietary intake of red meat, fish, cholesterol, egg, coffee, total fat and refined grains were adjusted in the different multivariable-adjusted models. The overall trend of ORs across increasing tertiles of DPI was examined by treating tertiles of DPI as ordinal variables. SPSS (SPSS Inc., version 19) was used for all statistical analyses. P-values of 0.05 were considered as significant.

## Results

**Fig. 1:**
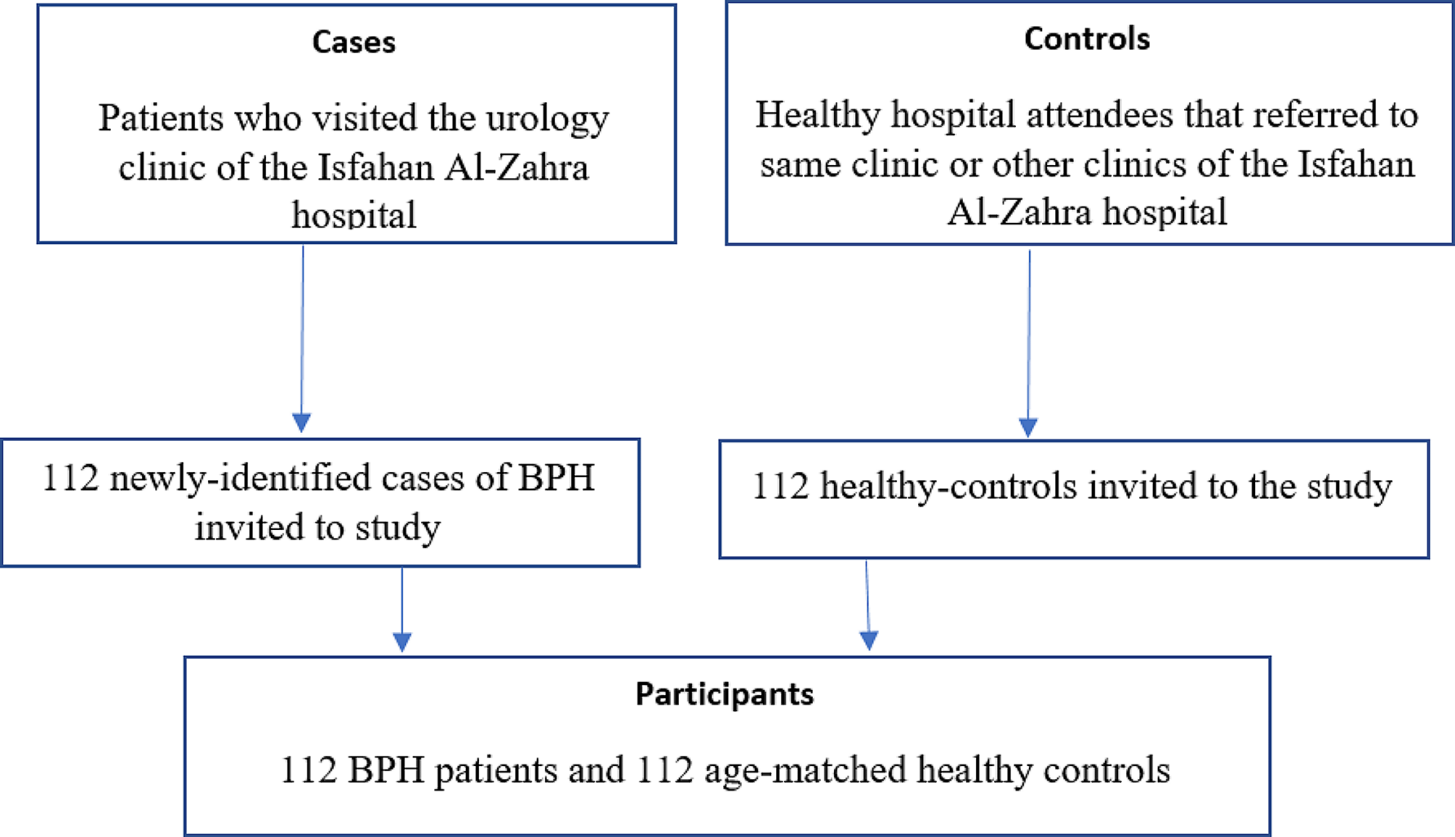
Flow diagram of the case-control study

Considering the possibility of dropouts, 224 people participated in this research. There were no dropouts. The study included 112 patients in the case group and 112 healthy individuals in the control group (Fig. 1). The characteristics of participants between groups and across tertiles of the Dietary Phytochemical Index are summarized in Table 1. The results showed that the overall DPI score was significantly higher in the controls than the cases (*p* = 0.01). Serum PSA levels were significantly higher in participants in the first tertile of DPI than the third tertile (*p* = 0.001).

**Table 1:** General characteristics of participants between groups and across tertiles of Dietary Phytochemical Index

variables^†^	Mean ± SD or N (%) Cases (*n* = 112)	Mean ± SD or N (%) Controls (*n* = 112)	*P*-value	Tertiles of DPI ( mean ± SD in all participants)			*P*-value^a^
				T1 ≤ 19	T2 19 < DPI < 28	T3 ≥ 28	
Age(year)	57.17 ± 8.04	58.2 ± 6.69	0.32	56.65 ± 7.32	58.08 ± 7.37	57.98 ± 7.58	0.62
Weight(kg) ^††^	72.8 ± 15.36	73.28 ± 11.97	0.8	74.38 ± 13.03	74.1 ± 13.93	70.6 ± 14.21	0.21
BMI(kg/m^2^)	25.56 ± 5	24.94 ± 3.69	0.32	25.69 ± 4.24	25.19 ± 4.31	24.89 ± 4.69	0.56
WC(cm) ^††^	95.6 ± 5.41	95 ± 4.26	0.32	95.92 ± 4.62	95 ± 5.01	95.34 ± 5.01	0.48
PSA (ng/mL)	6.17 ± 0.96	3.74 ± 0.67	**< 0.001**	5.51 ± 1.47	4.63 ± 1.35	4.81 ± 1.46	**0.001**
Total energy (Kcal) ^††^	2356.21 ± 673.67	2327.48 ± 658.89	0.76	2339.47 ± 520.13	2269.47 ± 636.65	2418.53 ± 808.24	0.43
DPI	25 ± 11	29 ± 10	**0.01**	15 ± 3	25 ± 3	40 ± 6	**< 0.001**
^‡^Categorical Variables							
Marital status, (%)							
Married	85(75.9)	92(82.1)	0.25	57(77)	60(80)	60(60)	0.87
Single	27(24.1)	20(17.9)		17 (23)	15 (20)	15 (20)	
Education (%)							
Under Diploma	28 (25)	29(25.9)	0.7	22(29.7)	17(22.7)	18 (24)	0.22
Diploma	37 (33)	39(34.8)		30(40.5)	20(26.7)	26(34.7)	
Bachelor	31(27.7)	33(29.5)		14(19.9)	26(34.7)	24 (32)	
Postgraduate	16(14.3)	11(9.8)		8(10.8)	12 (16)	7(9.3)	
Smoking status (%)							
Non smokers	58(51.5)	45(40.4)	0.11	49(65.7)	39 (51.5)	33(44.8)	**0.04**
Smokers	54 (48.5)	63 (56.6)		25 (34.3)	36(48.5)	41(55.2)	
Family history Of BPH							
Yes	62(55.4)	56(50)	0.42	40(54.1)	40(53.3)	38(50.7)	0.9
No	50(44.6)	56(50)		34(45.9)	35(46.7)	37(49.3)	
Physical activity							
Light	84(75)	90(80.4)	0.58	61(82.4)	62(82.7)	51(68)	**0.009**
Moderate	23(20.5)	19 (17)		11(14.9)	8(10.7)	23(30.7)	
Vigorous	5(4.5)	3(2.7)		2(2.7)	5(6.7)	1(1.3)	

^†^ Data are analyzed by two−sample t−test unless otherwise indicated ^††^ analysis performed by Mann −Whitney U test^‡^ Data are analyzed by chi−squared test or Fisher’s exact test. ^a^*P*−value Obtained from ANOVA or Kruskal –Wallis or Chi−square test, where appropriate. Bolded p-values (<0.05) denote statistical significance

As shown in Table 2, dietary fiber (*p* = 0.03), vitamin A (*p* = 0.02), E (*p* = 0.01), D (*p* = 0.001), folate (*p* = 0.002), selenium (*p* < 0.001), and magnesium (*p* = 0.001) intake was significantly higher in the controls compared to the cases. Participants in the third tertile of DPI consumed higher PUFA (*p* = 0.04), fiber (*p* < 0.001), vitamin A (*p* < 0.001), vitamin C (*p* < 0.001), folate (*p* < 0.001), beta-carotene (*p* = 0.001), zinc (*p* = 0.01) and magnesium (*p* < 0.001) in their diet compared with the first tertile. Dietary intake of vitamin D and E were significantly higher in the second tertile than others (*p* = 0.01 and *p* = 0.005, respectively).

**Table 2:** Nutrient intake of participants between groups and across tertiles of dietary phytochemical index

Nutrients†	Mean ± SD Cases (*n* = 112)	Mean ± SD Controls (*n* = 112)	*P*-value	Tertiles of DPI ( mean ± SD in all participants)			*P*-value^a^
				T1 ≤ 19	T2 19 < DPI < 28	T3 ≥ 28	
Carbohydrates intake(g/d)	344.21 ± 155.75	369.64 ± 156.92	0.22	331.46 ± 115.79	370.84 ± 171.64	368.13 ± 173.65	0.23
Protein intake(g/d)	92.48 ± 28.59	99.19 ± 20.02	0.24	89.09 ± 35.96	102.72 ± 50.94	95.61 ± 39.77	0.15
Total fat intake(g/d)	93.53 ± 38.8	94.37 ± 46.63	0.88	88.19 ± 33.96	100.02 ± 48.94	93.57 ± 43.8	0.24
Cholesterol (mg/d)	311.14 ± 181.32	361.95 ± 221.86	0.062	329.06 ± 164.37	372.93 ± 260.89	307.55 ± 167.75	0.13
Saturated fats(g/d)	28.67 ± 13.47	26.72 ± 12.73	0.26	27.1 ± 11.04	30.52 ± 16.96	25.46 ± 9.82	0.05
Monounsaturated fats(g/d)	30.92 ± 12.98	30.15 ± 16.21	0.69	29.34 ± 11.72	32.34 ± 17.56	29.92 ± 14.08	0.48
Polyunsaturated fats(g/d)	17.37 ± 10.89	19.07 ± 12.67	0.28	15.87 ± 7.44	18.09 ± 11.07	20.68 ± 15.24	**0.04**
Fiber (g/day)	19.37 ± 10.41	22.23 ± 10	**0.03**	16.78 ± 6.68	22.24 ± 11.26	23.32 ± 11.11	**< 0.001**
Vitamin A(RE)	1431.12 ± 1251.31	1895.53 ± 1422.01	**0.02**	1026.82 ± 6868.59	2027.8 ± 1727.23	1926.86 ± 18.02	**< 0.001**
Vitamin D(µg/d)	0.98 ± 1.02	1.49 ± 1.29	**0.001**	1.26 ± 1.26	1.51 ± 1.24	0.93 ± 1	**0.01**
Vitamin E(mg/d)	4.07 ± 2.31	5.84 ± 4.86	**0.01**	4.3 ± 2.82	6.15 ± 5.57	4.4 ± 2.18	**0.005**
Vitamin C (mg/d)	132.41 ± 83.74	151.96 ± 19.73	0.06	106.27 ± 49.95	154.25 ± 84.57	165.56 ± 85.08	**< 0.001**
Folate (mcg/day)	227.51 ± 145.05	340.7 ± 157.8	**0.002**	247.71 ± 104.6	334.97 ± 181.11	343.81 ± 150.57	**< 0.001**
Beta-carotene(µg/d)	636.47 ± 736.84	916.1 ± 654.2	0.07	390.34 ± 447.1	869.19 ± 562.95	1069 ± 985.18	**0.001**
Zinc(mg/d)	11.84 ± 5.66	6.23 ± 6.15	0.08	10.91 ± 4.71	13.69 ± 7	12.97 ± 5.58	**0.01**
Selenium (mg/d)	0.11 ± 0.05	0.14 ± 0.06	**< 0.001**	0.12 ± 0.05	0.13 ± 0.07	0.13 ± 0.06	0.85
Magnesium(mg/d)	306.23 ± 130.43	372.35 ± 172.98	**0.001**	272.83 ± 93.28	355.54 ± 153.27	388.61 ± 185.7	**< 0.001**
Iron(mg/d)	24.36 ± 12.63	24.14 ± 11.79	0.89	23.21 ± 10.95	24.95 ± 13.27	24.57 ± 12.32	0.66

^†^ data are analyzed by two−sample t−test. ^a^*P*−value Obtained from ANOVA test. Bolded p-values (<0.05) denote statistical significance

The dietary intakes of various food groups among participants categorized by DPI tertiles are provided in Table 3. Food group analysis showed that the BPH-patients consumed lower fish (*p* = 0.001), legumes (*p* < 0.001), and olives and olive oil (*p* = 0.02) in their diet compared to controls. Per day, all participants in the third tertile of DPI consumed higher fruits (*p* < 0.001), whole grains (*p* = 0.02), nuts (*p* < 0.001), legumes (*p* = 0.02), tomato and its products (*p* < 0.001), and olive (*p* = 0.02) than the first tertile. However, intake of refined grains was significantly higher in the first tertile of DPI (*p* < 0.001). Participants in the second tertile of DPI consumed higher red meat than the other tertiles, per day (*p* = 0.03).

**Table 3:** Dietary intakes of food groups of participants by tertiles of DPI

Food groups†	Mean ± SD Cases (*n* = 112)	Mean ± SD Controls (*n* = 112)	*P*-value	Tertiles of DPI ( mean ± SD in all participants)			*P*-value^a^
				T1 ≤ 19	T2 19 < DPI < 28	T3 ≥ 28	
Vegetables^1^ (g/day)	150.77 ± 94.52	162.54 ± 93.97	0.35	127.8 ± 67.61	175.52 ± 108.51	166.26 ± 95.8	**< 0.001**
Fruits^2^ (g/day)	211.49 ± 179.04	206.97 ± 131.1	0.82	133.28 ± 83.98	218.68 ± 158.53	274.7 ± 177.7	**< 0.001**
Whole grains^3^ (g/day)	50.51 ± 198.83	84.62 ± 118.48	0.12	29.17 ± 46.07	72.33 ± 103.34	100.69 ± 256.62	**0.02**
Refined grains^4^ (g/day)	410.21 ± 230.18	452.79 ± 246.37	0.18	516.01 ± 245.63	443.01 ± 240.52	336.6 ± 195.25	**< 0.001**
Nuts^5^ (g/day)	37.7 ± 53.78	31.48 ± 43.75	0.34	20.09 ± 17.35	29.16 ± 41.87	54.32 ± 67.49	**< 0.001**
Legumes^6^(g/day)	40.48 ± 29.13	58.7 ± 38.07	**< 0.001**	40.64 ± 30.04	54.02 ± 37.63	54 ± 35.66	**0.02**
High fat dairy products^7^ (g/day)	130.02 ± 135.55	112.02 ± 115.65	0.28	120.3 ± 59.13	155.28 ± 72	96.52 ± 38.28	0.66
Red meats^8^ (g/day)	209.59 ± 193.44	176.34 ± 143.54	0.14	172.47 ± 139.34	234.68 ± 123.17	171.48 ± 102.29	**0.03**
Fish^9^	5.18 ± 5.32	16.14 ± 18.84	**0.001**	9.75 ± 13.99	11.39 ± 15.39	10.84 ± 15.32	0.79
Tomato and its products	35.87 ± 28.65	42.45 ± 31.39	0.1	26.63 ± 19.09	45.21 ± 21.01	45.47 ± 24.24	**< 0.001**
Olives and olive oil	11.03 ± 26.84	28.04 ± 74.74	**0.02**	6.67 ± 4.23	20.25 ± 18.22	31.43 ± 17.32	**0.02**

^†^ Data are analyzed by two-sample t-test. ^a^*p*-value Obtained from ANOVA test. ^1^ Carrots, spinach, lettuce, eggplant, peppers, green beans, pumpkin, mushrooms, garlic, stewed vegetables, green beans, Cucumber, cabbage, peas. ^2^ Watermelon, melon, cantaloupe, apple, cherry, cherry, peach, nectarine, date, grape, kiwi, pomegranate, strawberry, banana, persimmon, berry, pineapple.^3^ Whole grain breads (Berberi, Sangak), barley, wheat and corn bread. ^4^ Lavash bread and baguettes, rice, pasta, flour, biscuits, Tafton bread. ^5^ Almonds, walnuts, peanuts, hazelnuts, pistachios. ^6^ Lentils, beans, chickpeas. ^7^ Full-fat milk, full-fat yogurt, cream cheese, cream, ice cream.8 Beef, lamb, minced meat. ^9^ all type of fishes, tuna. Bolded p-values (<0.05) denote statistical significance

Multivariable-adjusted ORs and 95% CI for BPH across tertiles of DPI are presented in Table 4. In the crude model, participants in the higher tertile of DPI had 70% lower odds of BPH compared to those in the lowest tertile (95% CI 0.15–0.61, *P*-trend = 0.001). After adjusting for energy intake and anthropometric measures, as the confounders, this inverse association remained strong (95%CI 0.14–0.58, *P*-trend = 0.001). In addition to the previous confounders, further adjustments were made for education level, family history of BPH, smoking, physical activity, marital status, and dietary intake. Despite these additional adjustments, the observed association between DPI tertiles and BPH remained unchanged (95%CI 0.15–0.63, P-trend = 0.001).

**Table 4:** Multivariable-adjusted odds ratios (95% CIs) for BPH across tertile categories of DPI

	Tertiles of DPI			*P*-trend
	T1 ≤ 19	T2 19 < DPI < 28	T3 ≥ 28	
**Crud Model**	1.00	0.21(0.1–0.4)	0.3(0.15–0.61)	0.001
**Model I**	1.00	0.21(0.1–0.44)	0.29(0.14–0.58)	0.001
**Model II**	1.00	0.18(0.08–0.39)	0.3(0.15–0.63)	0.001
**Model III**	1.00	0.15(0.06–0.36)	0.23(0.1–0.54)	0.001

Binary logistic regression was used to obtain OR and 95% CI. The overall trend of OR across increasing tertiles was examined by considering each category’s median score as a continuous variable. Model I: Adjusted for Energy intake, waist circumference, BMI, weight. Model II: Model I plus education level and family history of BPH, smoking status, physical activity, marital status. Model III: Model I and II plus red meat, fish, cholesterol, egg, coffee, total fat, refined grains

## Discussion

In this case-control study, high intake of dietary phytochemicals reported using the DPI index was inversely associated with the odds of BPH. This association remained significant after adjustment for several confounding variables including anthropometric measures, daily calorie and some food intake, physical activity levels and educational status. This study was the first investigation of the association between DPI and odds of BPH to date. Dietary patterns vary throughout the world [31], and intakes of refined carbohydrates and fats has increased mainly due to the industrialization in developing countries, a term called ‘nutrition transition’ [32]. These changes predispose a population to nutrition- and lifestyle- related chronic diseases at an exceeding rate [33]. BPH is a rising global condition involving 94 million men in 2019, compared with 51.1 million in 2000. It has particularly high prevalence and incidence in low- and middle-income countries due to rapid demographic and epidemiological changes. Increasing prevalence of BPH due to longer life expectancies emphasizes the need for continuous monitoring and proactive planning of healthcare systems to address the growing healthcare demands [34]. . More recent studies have focused on the association between DPI and risk of overweight and obesity, and metabolic disorders [35, 36], and despite an inverse association between DPI and some cancers such breast cancer and glioma in previous studies [37, 38], there is no study to assess the relationship between DPI and risk of BPH. A case-control study reported that women in the highest quartile of DPI had a 92% decrease in odds of developing breast cancer compared to women in the lowest quartile [37]. In the present study, participants in the higher tertile of DPI had 70% lower odds of BPH compared to those in the lowest tertile. One of the key mechanisms through which phytochemicals may contribute to cancer prevention is their antioxidant activity. Antioxidants help neutralize harmful free radicals and reactive oxygen species (ROS) in the body, which are known to cause DNA damage and promote cancer development [39] .In addition to their antioxidant properties, phytochemicals can also influence the activity of enzymes involved in the metabolism of carcinogens. Carcinogens are substances that can cause cancer, and their activation or detoxification largely depends on specific enzymes. Phytochemicals have been found to modulate the activity of these enzymes, particularly by inhibiting phase I enzymes such as cytochrome P450 and inducing phase II enzymes including glutathione S-transferases and UDP-glucuronosyltransferases [40]. Furthermore, the fiber content in a phytochemical-rich diet also contributes to cancer prevention. Dietary fibers, particularly soluble fibers found in fruits, vegetables, and whole grains, have been associated with a reduced risk of various types of cancer [41]. In the current study, healthy men consumed more fiber, vitamin A, E, D, folate, selenium, and magnesium in their daily diet compared with BPH patients. Higher intake of vitamin E, selenium, and magnesium has been linked to potential protective effects against BPH development, as these nutrients possess antioxidant and anti-inflammatory properties [42–44]. Participants in the third tertile of DPI consumed higher PUFA, fiber, vitamin A, vitamin C, folate, beta-carotene, zinc and magnesium compared with the first tertile. Herein, a diet with high DPI contained more vegetables, fruits, whole grains, nuts, legumes, olives and olive oil, tomato and its products but lower refined grains and red meat. In our study dietary intake of vitamin D, E and red meat were significantly higher in the second tertile than others. It is possible that individuals in the second tertile DPI may be choose to consume these foods or food containing this nutriens more frequently due to factors such as taste preferences, cultural practices, or individual dietary choices. This study marks the pioneering investigation of its kind, adding to the ever-growing body of evidence that supports the potential role of phytochemical-rich diets in preventing or managing BPH. These findings offer valuable insights for clinical practitioners and public health initiatives striving to alleviate the burden of BPH on individuals and society. However, previous studies have predominantly focused on examining isolated nutrients, rather than taking a comprehensive approach [45]. It is crucial to recognize that nutrients are not consumed in isolation, and the complex food matrix itself significantly influences nutritional outcomes. Consequently, relying solely on the effects of individual nutrients fails to provide a comprehensive explanation. Nutrients can exhibit additive or interactive effects, altering their actions when consumed together as part of a meal. The synergistic effects of phytochemicals present in diverse plant-based foods, consumed holistically, can profoundly impact metabolic outcomes [46]. Therefore, it is imperative to transcend the focus on individual nutrients and embrace a more holistic perspective within the Dietary Phytochemical Index (DPI) framework.

However, our study does have limitations. Self-reported dietary data is subject to recall bias, potentially impacting the accuracy of our findings. Cultural practices can influence dietary habits, potentially limiting the generalizability of our results beyond the Middle-Eastern population. Additionally, we lacked data on the severity of BPH. While we aimed to assess the association of DPI with the risk of BPH, we acknowledge the potential significance of evaluating the relationship with BPH severity using parameters such as prostate volume, International Prostate Symptom Score (IPSS), and uroflowmetry data. Future research endeavors should consider incorporating such measures to provide a more comprehensive understanding of the relationship between DPI and BPH, including its severity.

Regarding the selection of the control group, we opted for hospital controls. The criteria for their inclusion in the control group mirrored those applied to the cases, except for the absence of a BPH diagnosis. While we did not gather comprehensive details regarding the purpose of their hospital visit, our primary concern centered on verifying their eligibility for inclusion as controls. In consideration of these constraints, our study stands as an initial foray into investigating the plausible association between DPI and the risk of BPH.

## Conclusion

In conclusion, our study findings indicate a protective association between a higher DPI and the risk of BPH in men. This suggests that a diet rich in phytochemical-containing foods, such as whole grains, fruits and vegetables, and nuts, may contribute to a reduced likelihood of developing BPH among men. Therefore, it is crucial to emphasize strategies that improve physical and economic access to these healthy, phytochemical-rich foods, as they have the potential to mitigate the occurrence of common chronic diseases. Further research in this area is warranted to deepen our understanding of the mechanisms underlying the observed association and to explore additional factors that may influence the relationship between dietary phytochemicals and BPH risk.

## Data Availability

The datasets generated and analyzed during the current study are not publicly available due to our institute policies but are available from the corresponding author on reasonable request.

## Abbreviations

BPH: Benign Prostatic Hyperplasia
DPI: Dietary Phytochemical Index
PSA: Prostate-Specific Antigen
DRE: Digital Rectal Examination
FFQ: Food Frequency Questionnaire
IPAQ: International Physical Activity Questionnaire
OR: Odds Ratio
BMI: Body Mass Index
SFA: Saturated Fatty Acid
MUFA: Mono-Unsaturated Fatty Acid
PUFA: Poly Unsaturated Fatty Acid
IPSS: International Prostate Symptom Score
